# Comparative Side‐Effects of Neurosurgical Treatment of Treatment‐Resistant Depression

**DOI:** 10.1111/cns.70090

**Published:** 2024-10-28

**Authors:** Alexandre Lim Eng Keat, Keith Tan Jian Li, Teo Chuin Hau, Tomoko Soga

**Affiliations:** ^1^ Jeffrey Cheah School of Medicine and Health Sciences Monash University Malaysia Bandar Sunway Selangor Malaysia

**Keywords:** ablative surgery, deep brain stimulation, treatment‐resistant depression, vagus nerve stimulation

## Abstract

**Introduction:**

Treatment‐resistant depression (TRD) is a condition in which patients suffering from depression no longer respond to common methods of treatment, such as anti‐depressant medication. Neurosurgical procedures such as ablative surgery, deep brain stimulation, and vagus nerve stimulation have been used in efforts to overcome TRD.

**Objectives:**

This review aims to provide an overview of the side effects of neurosurgery performed in clinical studies related to depression.

**Methods:**

A literature search was conducted through PubMed, MEDLINE, EMBASE, Ovid, and ClinicalTrials.gov databases.

**Results:**

This review selected 10 studies for ablative surgery, 12 for deep brain stimulation, and 10 for vagus nerve stimulation, analyzing their side effect profiles of neurosurgery for TRD. The major side effects of each type of neurosurgery were identified, such as incontinence and confusion for ablative surgery, headaches and increased suicide ideation for deep brain stimulation, and voice hoarseness and dyspnea for vagus nerve stimulation.

**Conclusion:**

The review discusses the merits and demerits of neurosurgery as a treatment option for TRD. It also suggests new insights into decreasing the burden of these neurosurgical side effects so that they can be a viable, high‐efficacy treatment method for TRD.

## Graphical abstract

Neurosurgical treatments are used in the treatment of treatment‐resistant depression. The major side‐effects arising from each neurosurgical procedure differ depending on the region of the brain targeted by the treatment, with very little overlap between side‐effects. Increasing awareness of these potential side‐effects will assist clinicians in reducing the burden of treatment and provide patients with better quality of life.

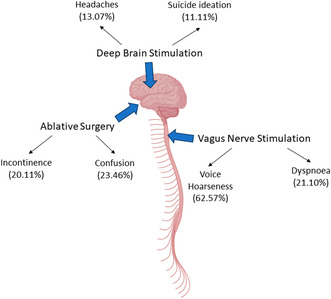

## 1 Introduction

Depression is a mood disorder commonly defined by a persistently low mood, feelings of sadness, and loss of interest in once enjoyable pursuits [1]. According to the established criteria in the Diagnostic and Statistical Manual of Mental Disorders (DSM‐V), patients suffering from depression exhibit symptoms such as affected sleep, reduction in interests, feelings of guilt, change in weight and energy, affected concentration and memory, psychomotor disturbances, and suicidal thoughts [2]. Studies performed across 30 countries have indicated that more than one in 10 people will experience depression at least once in their lives, further emphasizing the importance of proper mental health care [3].

Many patients may present with some or even all of the symptoms. These symptoms have often caused a reduction in the quality of life and even worsened the prognosis of many patients. Depression is also commonly seen together with other co‐morbidities such as anxiety disorders, as part of different mood disorders such as bipolar disorder, or even as part of an organic disease such as hypothyroidism. Thus, it is essential to rule out other causes of depression in patients who present with depressive symptoms, as well as to assess their risk of harm to themselves and others.

Currently, depression is commonly treated with a standard regime of medication, lifestyle modification, and psychotherapy [1]. As clinical studies have shown there has been involvement of serotonin (5‐HT) receptors in the CNS, serotonin selective reuptake inhibitors (SSRIs) have been proven to be an effective modality in the treatment of depression [4, 5]. Other medications such as serotonin‐norepinephrine reuptake inhibitors (SNRIs), monoamine oxidase inhibitors (MOA‐i), and tricyclic antidepressants (TCAs) have also been shown to help in patients who do not respond to the typical first‐line medication [4, 5]. Antidepressants have been demonstrated to reduce the rate of relapse by an average of 27% [6].

Despite the options for treatment available, there are patients with depression who reach a stage where the disease continues to progress. For these patients, the term “treatment‐resistant depression” (TRD) has been set to identify patients who will be at further risk of having a poorer prognosis, as well as attempting to identify further modalities to allow treatment for this group of patients. It has been suggested that TRD is set when a patient has failed two antidepressant medication trials of 6–8 weeks at adequate doses despite full compliance [7]. TRD approximates 30% of all patients diagnosed with major depressive disorder and continues to remain a major burden not only to healthcare due to the cost of inpatient care and treatment cost but also socioeconomically as billions of dollars are lost due to unemployment and productivity burden [8, 9]. A study has shown that the incremental economic impact of Major Depressive Disorder (MDD) in society was calculated to be $333.7 billion in 2023 US dollars, equating to $16,854 per adult affected by MDD. The primary factors driving these costs included healthcare expenses, household‐related costs, presenteeism, and absenteeism [10].

Patients with TRD are generally offered an augmentation with antipsychotics on top of an existing antidepressant or electroconvulsive Therapy (ECT), with a response rate of 65.9%, as the next line of management. However, should this be contraindicated or unsuccessful, other treatment modalities, such as neurosurgery, may be explored [11].

Ablative surgery (AS), also known as destructive surgery, can be performed intentionally to destroy specific areas of brain tissue or to disrupt certain neural circuits [12]. Ablative surgery is done initially by marking specific target lesions in the brain, followed by either radiofrequency thermal coagulation, cryotherapy—which induces tissue death by freezing, radiosurgery, or laser surgery [12, 13]. Examples of ablative surgery done in the brain include anterior cingulotomy, which destroys cortical tissue; anterior capsulotomy, which targets the anterior limb of the internal capsule, disrupting the communication between the thalamus and the cortex; subcaudate tractotomy, which interrupts the tracts between the orbitofrontal cortex and subcortical limbic structures; and finally the limbic leucotomy, which is a combination of anterior cingulotomy and subcaudate tractotomy [12, 13]. Each of these procedures aims to disrupt specific brain regions or pathways that are implicated in the pathophysiology of depression. Recent advances in neuroimaging and surgical techniques, such as MRI‐guided focused ultrasound (MRgFUS) and gamma knife radiosurgery, have improved the precision and safety of these procedures, enhancing their therapeutic potential while minimizing risks [14]. These advancements have also spurred renewed interest in the use of ablative surgery for psychiatric disorders, including TRD [14].

Deep brain stimulation (DBS), as the name suggests, is when the deeper parts of the brain are stimulated with an electrode. This method has been in practice since the 1950s, allowing humans to have their brains chronically stimulated through metal electrodes [15, 16]. The surgery is done by inserting a stimulator, an extension, and electrodes placed onto certain brain areas. This would include the superolateral branch of the medial forebrain bundle, the ventral anterior limb of the internal capsule, the nucleus accumbens, and the subcallosal cingulate gyrus [17]. These structures are implicated in mood regulation and emotional processing, making them ideal targets for TRD. Many studies have been conducted to determine the superiority of locations. However, there has not been much conclusive evidence. The application of DBS extends to conditions beyond TRD, such as Parkinson's disease, epilepsy, depressive disorders, OCD, Tourette's syndrome, anorexia nervosa, and even addiction medicine [17].

Vagus nerve stimulation (VNS) is similar to DBS. A lead wire is coiled around the vagus nerve, and a stimulator, implanted at the left chest, sends our regular impulse signals to the brain through the cranial nerve [18]. The exact mechanism of this neurosurgical technique is still not known. A proposed hypothesis suggests that VNS may induce desynchronization of neuronal activity through the modulation of neurotransmitter release, impact hippocampal plasticity, and exhibit anti‐inflammatory effects. Additionally, long‐term VNS is believed to influence the neurotransmission of key substances such as norepinephrine, serotonin, and gamma‐aminobutyric acid (GABA), which can help alleviate depressive symptoms in patients who do not respond to conventional treatments [19, 20]. However, it is effective in the treatment of refractory epilepsy and has seen an increasing number of uses in recent times for depressive disorders, including TRD [18].

Since neurosurgical treatment for depression may have potentially unwanted outcomes thus far, a review of the side effects of neurosurgical treatment performed in clinical studies related to depression may be of use in discussing the merits and demerits of neurosurgery as a treatment option for depression.

## 2 Methods

This study has been carried out in multiple stages. A literature review was conducted after learning more about the status quo of TRD. An array of databases, including PUBMED, MEDLINE, EMBASE, Ovid, and ClinicalTrials.gov, have been consulted and explored to obtain and retrieve relevant studies required for this study.

The search criteria were categorized into three types of searches: ablative surgery, deep brain stimulation, and vagus nerve stimulation.

For ablative surgery, keywords included are (“treatment‐resistant depression” OR “treatment‐refractory depression”) AND (“anterior cingulotomy” OR “anterior capsulotomy” OR “limbic leucotomy”). No advanced search criteria were added to further filter the studies due to the low number of studies returned. A total of 47 studies were returned to be assessed for eligibility. From these studies, 10 were eligible to be included in the review as they fulfilled all search criteria and contained side‐effect profiles as data.

For deep brain stimulation, keywords included are “treatment‐resistant depression” OR “treatment‐refractory depression” AND “deep brain stimulation” NOT (“Parkinson's disease” OR “epilepsy”). This initial search yielded around 1120 studies. The filters (“humans,” “English,” and “past 20 years”) were used to reduce the number of studies further. A total of 244 studies were returned to be assessed for eligibility. From these studies, 12 were eligible to be included in the review as they fulfilled all search criteria and contained side‐effect profiles as data.

Finally, for vagus nerve stimulation, keywords included are (“treatment‐resistant depression” OR “treatment‐refractory depression”) AND (“vagus nerve stimulation” OR “vagal nerve stimulation”). From this initial search, around 2008 studies were returned. The filters (“humans,” “English,” and “past 20 years”) were used to filter the search. A total of 136 studies were returned to be assessed for eligibility. From these studies, 10 were eligible to be included in the review as they fulfilled all search criteria and contained side‐effect profiles as data.

## 3 Results

The ablative surgery procedures performed were primarily bilateral anterior capsulotomies and anterior cingulotomies, with a single study on the subcaudate tractotomy procedure. The studies of ablative surgery spanned periods ranging from 28 months to 6 years. Every study besides one reported side effects, with most being potentially severe, such as headaches, hallucinations, cognitive impairment, and memory difficulties (Table 1). All but one of the studies were also performed on mixed‐gender cohorts, with only one study focusing on solely female patients [25]. The full breakdown of side effects in each study is presented in Table 2. Confusion has the highest occurrence rate across all studies (23.46%), followed by urinary incontinence (20.11%). One study in particular appears to have had half of the patients report both symptoms [23]. Other noteworthy symptoms were headaches (17.88%) and fatigue (11.17%) (Table 1).

**TABLE 1:** Side‐effects presented in ablative surgery studies.

References	[18]	[21]	[22]	[23]	[24]	[25]	[26]	[27]	[28]	[29]	Total	%
Urinary incontinence				24			3	1	4	4	36	20.11
Urinary retention					1						1	0.56
Nausea					1				5		6	3.35
Dizziness							2		2		4	2.23
Headache	1	1			7		8		15		32	17.88
Confusion		3		24	2	2	5		6		42	23.46
Ataxia							1				1	0.56
Concentration problems				4	1		2				7	3.91
Memory problems		3		10	1		2			1	17	9.50
Amotivation		1		6	1	1	1				10	5.59
Tiredness/Fatigue		1		14	1	1	3				20	11.17
Somnolence							1				1	0.56
Seizures/Epilepsy				1	1		1		1	1	5	2.79
Weight gain				3	1		3				7	3.91
Subjective personality change				2			2				4	2.23
Lack of emotional response						2	1				3	1.68
Addiction							1				1	0.56
Pin‐site swelling	1				1						2	1.12
Visual pseudo‐hallucinations		1				1					2	1.12
Increased appetite		1				1					2	1.12
Increased libido		1									1	0.56
Decreased smell/taste		1				1					2	1.12
Difficulty forming higher order thoughts and idea		2				1					3	1.68
Psychomotor slowing		3									3	1.68
Facial weakness		2									2	1.12
Infection				1							1	0.56
Facial swelling									5		5	2.79
Intracerebral hemorrhage									1		1	0.56
Involuntary limb movements										1	1	0.56
Intracranial abscess										1	1	0.56

*Note:* Side‐effects are listed as each incident reported in each study referenced. The occurrence of side effects is presented as a percentage of participants reporting the side effect amongst the total participants. Participant total: *n* = 179.

**TABLE 2:** Side‐effects presented in deep brain stimulation studies.

References	[30]	[31]	[32]	[33]	[34]	[35]	[36]	[37]	[38]	[39]	[40]	[41]	Total	%
Suicide ideation/attempt	2		3	1		2			7	2	1		17	11.11
Insomnia	4					1		1					6	3.92
Anxiety/Aagitation	2				1			2			2		5	3.27
Change in smell/taste	1												1	0.65
Tearfulness	2												2	1.31
Hot sensation/physical discomfort	2												2	1.31
Dizziness	1						6						7	4.58
Involuntary muscle movement	1												1	0.65
Nausea/Vomiting	1					9							10	6.54
Diarrhea	1					1							2	1.31
Pain (surgical site)	2			2	1						1		5	3.27
Surgical site infection		2			4								6	3.92
Worsening depression			3							1			4	2.61
Seizure/Epilepsy					1				1				2	1.31
Headache					4	6	6		4		1	8	20	13.07
Chest pain						1							1	0.65
Pneumonia						1							1	0.65
Infection						1							1	0.65
Musculoskeletal (spasm, tremor, stiffness)						4							4	2.61
Itching						6							6	3.92
Polyuria						2							2	1.31
Weight gain						1							1	0.65
Tinnitus						1			2				3	1.96
Rash									2				2	1.31
Eyelid swelling									3				3	1.96
DVT/PE									1				1	0.65
Syncope										1			1	0.65
Dyspnoea										1	1		1	0.65
Hyperkinesia											1		1	0.65
Poor wound healing											2		2	1.31
Vision disorder (blurred vision/strabismus/diplopia)											16		16	10.46
Hypomania											1	8	9	5.88
Hypertension											4		4	2.61

*Note:* Side‐effects are listed as each incident reported in each study referenced. The occurrence of side effects is presented as a percentage of participants reporting the side effect amongst the total participants. Participant total: *n* = 153.

The clinical studies on deep brain stimulation are summarized in Table 2, which also presents the breakdown of the side effects in each study. The studies spanned periods ranging from 28 months to more than 6 years. Most of the studies in this category stimulated the subcallosal cingulate gyrus or the subgenual cingulate. There were also studies on the stimulation of the nucleus accumbens, the ventral anterior limb of the internal capsule, and the superolateral medial forebrain bundle, respectively; of these, the Coenen study, which stimulated the superolateral medial forebrain bundle, reported unique side‐effects, namely hyperkinesia, poor wound healing, and strabismus/diplopia [40]. The distribution of side effects appears to be more widespread and less prevalent in deep brain stimulation studies. Compared to ablative surgery, the major side effects are different, likely due to the different locations treated in the respective procedures. Headaches have the highest occurrence rate across all studies (13.07%), followed by suicide ideation/attempts (11.11%) and strabismus/diplopia (10.46%) (Table 2).

The vagus nerve stimulation studies are summarized in Table 3. The time range for the studies was from 18 months to more than 27 years post‐surgery. All of the studies were conducted on both genders. As with ablative surgery and deep brain stimulation studies, the vagus nerve stimulation studies were conducted on patients in their late 40s or 50s. Two studies had large cohorts of 331 and 205 patients, respectively [49, 51], resulting in a far larger number of total participants recorded in this review than the other two procedures. Except for the Evensen study [43], every other study in the review reported voice hoarseness as a side effect. Voice hoarseness has the highest occurrence rate across all studies (62.57%), followed by cough (22.54%), dyspnea (21.10%), pain at the surgical site (15.32%), and neck pain (14.6%).

**TABLE 3:** Side‐effects presented in vagus nerve stimulation studies.

References	[42]	[43]	[44]	[45]	[46]	[47]	[48]	[49]	[50]	[51]	Total	%
Voice hoarseness	3		7	4	4	17	6	239	18	135	433	62.57
Neck pain	2	1		1	3		6	46	15	27	101	14.60
Cough	2		5		3			83	8	55	156	22.54
Nausea/Vomiting	1							39		13	53	7.66
Arm myoclonus	1										1	0.14
Hypomania	1										1	0.14
Dizziness	1										1	0.14
Anxiety		1			1			38			40	5.78
Worsening depression		1		3				60	13	15	92	13.29
Feeling of vibration		1									1	0.14
Tiredness/Fatigue		1		1							2	0.29
Headache		3			2			61		12	78	11.27
Skin wound at electrode placement area		1									1	0.14
Erythema of skin at electrode placement area		1									1	0.14
Postauricular numbness			1								1	0.14
Dyspnoea				1				107	4	34	146	21.10
Decreased appetite				1							1	0.14
Paraesthesia					1			8		26	35	5.06
Pain (surgical site)							1	105			106	15.32
Insomnia								36		10	46	6.65
Attempted suicide/ideation									2	3	5	0.72
Mania									1	2	3	0.43
Dysphagia										31	31	4.48

*Note:* Side‐effects are listed as each incident reported in each study referenced. The occurrence of side effects is presented as a percentage of participants reporting the side effect amongst the total participants. Participant total: *n* = 692.

## 4 Discussion

This review presents the side effects of three different variants of neurosurgery for treatment‐resistant depression: (1) ablative surgery, (2) deep brain stimulation, and (3) vagus nerve stimulation. Although these treatments are highly effective, they may also produce serious side effects and changes in brain functions.

Ablative surgery may be presented as an alternative option if patients exhibit any of the specified relative contraindications or demonstrate an inability to tolerate the potential side effects associated with ECT. The average response rate of ablative surgery is roughly 40% [52]. However, it is worth noting that ablative surgery also carries its own side effects. Of the symptoms that occur, we see that urinary incontinence, confusion, and memory loss occur more in ablative surgery (Table 1). With regards to urinary incontinence, this is most likely due to the close relationship of the ablative site and the micturition center (pontine micturition center) and the hippocampus. The anterior cingulate cortex plays a role in some aspects of bladder control [53]. Confusion and memory loss may be due to the role that the anterior limb of the interior capsule plays in cognition [54]. While these side effects are commonly characterized as “transient,” their potential severity cannot be understated, warranting comprehensive preoperative patient counseling. Patients need to be informed about the possibility of requiring adult diapers or assistance from a caretaker during the initial post‐surgical period. This is crucial for managing expectations and ensuring adequate support during the recovery phase. Comprehensive support for patients undergoing ablative surgery encompasses various facets. Rehabilitation services, including physical and occupational therapy, are instrumental in assisting patients to regain independence and manage functional challenges post‐surgery. Ongoing psychosocial support, provided by mental health professionals and through support groups, addresses the psychological aspects of recovery and aids patients in coping with the transitional period. Assessing the need for home care services ensures a conducive environment during the immediate post‐surgical phase, offering vital assistance with daily activities.

DBS has undergone a thorough investigation as a treatment avenue for individuals with TRD. Across various targeted brain regions, DBS demonstrates an average response rate of approximately 60% in patients with chronic and previously refractory depression [55], an increase over that of ablative surgery. Nevertheless, there is substantial variability in response rates amongst patients and across different studies, often necessitating extensive experimentation and adjustment of stimulation parameters through trial‐and‐error methods to optimize therapeutic outcomes. As per the findings presented in Table 2, suicide ideation and attempts have been reported in half of the studies found, making up 11.1% of the overall patient number. It is a significant adverse event of DBS that should be given due attention. It is unclear whether DBS directly influenced their suicidality or if, for these patients, their depression had worsened to the point of them feeling this way. The prevalence of suicide ideation in DBS patients post‐procedure is known; however, while there has been discourse and debate on the putative causes, the causal associations are yet to be clearly demonstrated [56]. Regardless, it is essential that the risk of suicide after DBS is not dismissed and proper support is provided to the patients as part of their aftercare. Thus, patients with DBS should have closer monitoring, stringent follow‐up, and close participation of their family members in the aftercare process. It is important to utilize standardized suicide risk assessment tools such as the Columbia‐Suicide Severity Rating Scale (C‐SSRS) or the Suicide Ideation Questionnaire (SIQ) to quantify the severity of suicidal ideation and inform the immediate response. In the presence of signs of suicidal ideation, it is imperative to respond promptly by initiating a crisis intervention plan, involving the patient's support system, and documenting the incident thoroughly for future reference. Coordinating with mental health professionals and emergency services is vital to ensure a thorough and supportive response [57]. Another common side effect reported in DBS is strabismus or diplopia. This is a well‐recorded side effect due to the region of the brain commonly selected for the DBS procedure [58], and patients should be monitored after the procedure to ensure it does not interfere with their quality of life.

The response rates for patients with TRD who have undergone VNS are estimated to be around 80% a year after the procedure [59]. Within the studies compiled in this review, voice hoarseness is present in more than half of the participants (62.57%), with a smaller but still significant 20% of the patients experiencing cough and dyspnea (Table 3). These side effects are well‐recorded and expected. This is due to the procedure potentially causing downstream stimulation of the recurrent laryngeal nerve, a branch of the vagus nerve [44]. The cough reflex is also stimulated through the vagus nerve, causing patients to cough [60]. These side effects diminish with time and can be treated with common medication.

Headache and surgical site pain are commonly seen in all three neurosurgical treatments. Post‐surgery analgesia should be adequate, and patients who are experiencing pain may bring this up to their doctor to escalate their pain medication. Generally, simple analgesics such as paracetamol will be used initially, escalating to COX‐2 inhibitors such as Celebrex and then NSAIDs if needed. Opioids should be avoided unless patients fail to have pain relief even after trying NSAIDs. There may be other, more serious, surgical complications, such as intracerebral hemorrhages [28] and intracerebral abscesses [29], as seen in Table 1. While only one case of each side effect happened in these studies, these are still major complications that clinicians should be careful about. These may occur on top of infection of the wound site and reinsertion of the stimulation equipment, resulting in further injury [34, 35]. Ultimately, these occurrences, while rare, suggest a need for more excellent care in the execution of these procedures.

Future research should aim to make neurosurgical treatments for TRD more precise and safer. Advancements in imaging technology and surgical techniques could reduce side effects and improve patient outcomes. For example, more accurate targeting of brain regions during procedures could lessen damage to surrounding tissues. Additionally, long‐term studies are needed to understand how effective and safe these treatments are over time.

Integrating psychosocial support and rehabilitation services into standard care protocols is also essential for managing the side effects of such treatment modalities. These services can help in the overall recovery of patients, addressing not just the neurological but also the psychological and social aspects of their well‐being. Comprehensive care can assist patients in coping with the challenges posed by their condition and the treatments.

## 5 Conclusion

As presented in this review, common side effects of neurosurgical treatment include transient memory impairment, temporary cognitive effects such as confusion, and physical symptoms like headaches and muscle soreness. In more severe cases, there may also be intracranial bleeding or abscesses, as well as suicide attempts. A lot of uncertainty still exists as to why particularly severe, unwanted side effects such as suicide ideation occur as a result of neurosurgical treatment for depression. In TRD patients, maladaptive changes in damaged nerves manifest as side effects, leading to chemical, structural, and functional changes in the brain. These effects can persist for a prolonged period after the direct impact of the neurosurgical procedure itself has been resolved. Various preoperative endogenous factors such as genetics, age, and gender in TRD may be a predictor of poor outcomes of neurosurgery, inducing unwanted side effects.

In order to minimize the impact of these side effects, there needs to be a greater understanding of the safety profile of these procedures. The selection of a suitable treatment modality should be guided by considerations such as the patient's profile, individual tolerance to potential side effects, personal preferences, and the anticipated success rate of the chosen intervention. The suitability of alternative treatment modalities for TRD becomes particularly relevant when neurosurgical treatment is contraindicated or ineffective. For example, neurosurgical treatment may be contraindicated for individuals with cardiovascular issues, intracranial masses, recent strokes, certain medical conditions, and severe osteoporosis due to associated risks. While the field of neurosurgical treatment for depression has existed for many decades at this point, much remains to be discovered. More studies looking into the side effects and adverse events of neurosurgical treatments for TRD in the peri‐operative period should be carried out in order to increase our understanding of the side effects and improve our ability to select suitable treatment methods for the patients, even as the potential of these treatments is further fulfilled by technological advances.

## Conflicts of Interest

The authors declare no conflicts of interest.

## Supporting information


Data S1.

Table S1.

## Data Availability

The data that supports the findings of this study are available in the Supporting Information of this article.
