# Supplementary material for: Comparative Side‐Effects of Neurosurgical Treatment of Treatment‐Resistant Depression
Source: CNS Neurosci Ther. 2024 Oct 28;30(10):e70090. doi: 10.1111/cns.70090 (PMC11518690; doi:10.1111/cns.70090)
Supplement: Supplementary file 1 — Data S1. [file CNS-30-e70090-s001.zip › PRISMA_2020_flow_diagram_AS.docx]

**Ablative Surgery**

**Identification of studies via databases and registers**

Records removed *before screening*:

Duplicate records removed (n = 2 )

Records marked as ineligible by automation tools (n = 0 )

Records removed for other reasons (n = 0 )

Records identified from*:

Databases (n = 57)

Registers (n = 0)

**Identification**

Records screened

(n = 55 )

Records excluded

(n = 8 )

Reports sought for retrieval

(n = 47)

Reports not retrieved

(n = N/A)

**Screening**

Reports assessed for eligibility

(n = 47 )

Reports excluded:

Not in English (n = 4 )

Reviews (n = 8 )

Does not fit the review criteria (n = 25 )

Studies included in review

(n = 10 )

**Included**
