# Supplementary material for: Comparative Side‐Effects of Neurosurgical Treatment of Treatment‐Resistant Depression
Source: CNS Neurosci Ther. 2024 Oct 28;30(10):e70090. doi: 10.1111/cns.70090 (PMC11518690; doi:10.1111/cns.70090)
Supplement: Supplementary file 2 — Table S1. [file CNS-30-e70090-s002.docx]

Table 1S: Summary of Ablative Surgery Studies

| No | Citation | Type of surgery | n | Age (mean) | Gender | Depression duration | Country | Side effects |
| --- | --- | --- | --- | --- | --- | --- | --- | --- |
| 1A | Davidson, B. et al. 2020 (17) | Bilateral Anterior Capsulotomy | 16 | 41 | Mix | 16 years | Canada | Erythema at frontal pin sites, headache, Supraorbital pin-site swelling |
| 2A | Avecillas-Chasin, JM. et al.  2020 (20) | Bilateral Anterior Capsulotomy | 10 | 40.9 | Mix | N/A | USA | Confusion, visual pseudohallucination, increased appetite, decreased motivation, decreased smell, increased libido, fatigue, emotional blunting, difficulty forming higher order thoughts, poor memory, poor grooming, headache, psychomotor slowing, facial weakness |
| 3A | Martínez-Álvarez, R.  2019 (21) | Anterior Cingulotomy | 3 | 44 | Mix | N/A | Spain | No side effects |
| 4A | Subramanian, L. et al.  2016 (22) | Bilateral Anterior Capsulotomy | 45 | 45.5 | Mix | 20 years | UK | Focal seizures, urinary incontinence, fatigue and weight gain |
| 5A | Christmas, D. et al.  2015 (23) | Anterior Cingulotomy / Anterior Capsulotomy | 10 | 42.5 | Mix | Since 27.5 years old | UK | Headache, confusion, seizure, intermittent troublesome nausea, urinary retention, concentration and memory difficulties |
| 6A | Hurwitz, TA. et al. 2012 (24) | Bilateral Anterior Capsulotomy | 7 | 42.5 | Female | 6 years | Canada | Postoperative confusion, visual pseudohallucinations, increased appetite, decreased motivation, decreased smell and taste, increased libido, fatigue, subjective emotional blunting, difficulty formulating higher-order thoughts and ideas, fatigue |
| 7A | Christmas, D. et al.  2010 (25) | Bilateral anterior capsulotomy | 20 | 37.8 | Mix | Since 37.8 years old | UK | Urinary incontinence, nausea, dizziness, headache, confusion, ataxia, concentration probles, memory problems, amotivation, somnolence, tiredness, weight gain, seizures/epilepsy, weight gain, subjective personality change, lack of emotional response, addiction |
| 8A | Kim M.C. et al  2008 (26) | Subcaudate tractotomy | 10 | N/A | N/A | N/A | Korea | Mild transient urinary incontinence |
| 9A | Eljamel M. S. et al 2008 (27) | Bilateral anterior capsulotomy | 25 | 43 | Mix | 10.5 years | UK | Headache, nausea, dizziness, incontinence, confusion, facial swelling, seizure, intracerebral haemorrhage |
| 10A | Shields D.C. et al 2008 (28) | Anterior cingulotomy | 33 | 43.2 | Mix | N/A | USA | Transient urinary incontinence, involuntary limb movements with speech difficulty, memory impairment, new onset tonic-clonic seizures, intracranial abscess |

Table 2S: Summary of Deep Brain Stimulation Studies

| No | Citation | Location of stimulation | n | Age (mean) | Gender | Depression duration | Country | Side effects |
| --- | --- | --- | --- | --- | --- | --- | --- | --- |
| 1B | Fitzgerald et al. 2018 (29) | Nucleus accumbens | 5 | 44.6 | F | 6.5 years | Australia | Insomnia, anxiety/agitation, change in taste, tearfulness, hot sensation/physical discomfort, dizziness, involuntary smile, nausea, diarrhea, pain |
| 2B | Mayberg H.S. et al  2005 (30) | Subgenual cingulate | 6 | 46 | Mix | 5.6 years | Canada | Surgical site infection |
| 3B | Kennedy S.H. et al 2011 (31) | Subcallosal cingulate gyrus | 8 | 47.4 | Mix | 6.9 years | Canada | Hospitalisation (worsening depression, suicidal ideation), aggressive behaviour, lack of functional integration into workforce |
| 4B | Puigdemont D. et al 2011 (32) | Subcallosal cingulate gyrus | 20 | 35 | Mix | 6.3 years | Spain | Cephalalgia, pain in the neck at the site of the subdermal cable |
| 5B | Lozano A. M. et al  2008 (33) | Subcallosal cingulate gyrus | 21 | 47.4 | Mix | 6.8 years | Canada | Wound infection and hardware removal, reinsertion of DBS hardware, wound infection managed with antibiotics alone, perioperative seizure, worsening mood/irritability, perioperative headache, pain at pulse generator site |
| 6B | Lozano A. M. et al 2012 (34) | Subcallosal cingulate gyrus | 6 | 47.3 | Mix | 5 years | Canada | Skin erosion, extension break, chest pain, pneumonia, infection, suicide attempt, nausea, vomitting, diarrhoea, tremor, spasms, stiffness, superficial cellulitis, wound drainage, headache, persistent pain, psychiatric changes, dizziness, polyuria, weight gain, tinnitus, insomnia |
| 7B | Merkl A. et al  2013 (35) | Subgenual cingulate gyrus | 4 | 50.6 | Mix | Since 23.6 years old | Germany | Headaches, pain and scalp tingling at the surgical site, dizziness and sore throat due to anesthesia |
| 8B | Ramasubbu R. et al  2013 (36) | Subcallosal  cingulate gyrus | 16 | 50.2 | Mix | 6.4 years | Canada | Insomnia, anxiety, confusion and drowsiness |
| 9B | Alemany C. et al 2023 (37) | Subcallosal  cingulate gyrus | 25 | 46.5 | Mix | 28.2 months | Spain | Rash, spinal arachnoid cyst, senile cognitive impairment, neuroleptic-induced movement disorders, focal epilectic seizures, restless legs syndrome, headache, mild renal insufficiency, UTI, eyelid edema/cellulitis/ptosis, pulmonary embolism, DVT, IDA |
| 10B | Bergeld I.O. et al 2022 (38) | Ventral anterior limb of internal capsule | 8 | 53.2 | Mix | 83 months | Netherlands | Suicidal ideation/attempt, syncope, possible seizure, dyspnea after IPG replacement, relapse in depression after IPG replacement |
| 11B | Coenen V.A. et al  2019 (39) | Superolateral medial forebrain bundle | 16 | 51.6 | Mix | N/A | Germany | Blurred vision, and double vision, restlessness, slurred speech in one patient, hyperkinesia, Severe wound healing disturbances |
| 12B | Merkl A. et al  2018 (40) | Subcallosal cingulate gyrus | 8 | 48.2 | Mix | 28 months | Germany | Headaches, pain and scalp tingling at the surgical site, dizziness, light hypomania |

Table 3S: Summary of Vagus Nerve Stimulation Studies

| No | Citation | n | Age (mean) | Gender | Depression duration | Country | Side effects |
| --- | --- | --- | --- | --- | --- | --- | --- |
| 1C | Pigato G. et al.  2023 (41) | 6 | 48 | Mix | 2 years | Italy | Hoarseness, neck pain, cough, nausea and vomiting, arm myoclonus, hypomania, extrapyramidal symptoms, dizziness |
| 2C | Evensen K. et al 2022 (42) | 20 | 49.4 | Mix | 31.5 months | Denmark | Feeling of depression and exhaustion, vibration reminding of side effect of earlier electroconvulsive treatment and crippling sensations, and flashbacks, tiredness and strange feelings in the body, pain in neck and pressure in the head and more appetite and anxiety of unusual kind, headache, Skin wound at electrode placement area, redness of skin at electrode placement area |
| 3C | Zhang X. et al  2022 (43) | 7 | 45.7 | Mix | 17 years | China | Voice alteration, Cough, throat discomfort, postauricular numbness |
| 4C | Longpre-Poirier C.L. et al  2021 (44) | 14 | 47.6 | Mix | N/A | Canada | Loss of voice alteration, decreased perceptible stimulation and muscles contractions, pain and dyspnea, stridor and associated pain irradiation to the shoulder, neck and forearm |
| 5C | Albert U. et al  2015 (45) | 5 | 56.6 | Mix | 18.4 months | Italy | Hoarseness, neck pain, sore throat, headache, paresthesia, anxiety, dysphagia |
| 6C | Tisi G. et al  2014 (46) | 27 | 57.5 | Mix | 18.5 years | Italy | Voice alteration/hoarseness |
| 7C | Dell’Osso, B. et al  2013 (47) | 6 | 48.6 | Mix | Since 29 years old | Italy | Hoarseness, hypophonia, cough, and neck pain |
| 8C | Aaronson S.T. et al  2013 (48) | 331 | 47.9 | Mix | 27.0 years | USA | Voice alteration, dyspnea, pain, paresthesia, incision pain, increased cough, headache, depression, hypertonia, neck pain, dysphagia, nausea, anxiety, insomnia, device site reaction |
| 9C | Bajbouj M  2010 (49) | 74 | 47.4 | Mix | 3.5 years | Belgium, Germany, Ireland, Sweden, Switzerland, UK | Voice alteration, cough, pain and dyspnea |
| 10C | Rush A. J. et al 2005 (50) | 205 | 46.3 | Mix | 49.9 months | USA | Headache, neck pain, pain, dysphagia, nausea, insomnia, paresthesia, cough increased, dyspnea, laryngismus, pharyingitis, voice alteration, mania, suicide attempts, worsening depression, hospitalizations |
